# Taz/Tead1 Promotes Alternative Macrophage Activation and Kidney Fibrosis via Transcriptional Upregulation of Smad3

**DOI:** 10.1155/2024/9512251

**Published:** 2024-07-30

**Authors:** Yizhi Ren, Lu Zhou, Xinyuan Li, Xingwen Zhu, Zhiheng Zhang, Xiaoli Sun, Xian Xue, Chunsun Dai

**Affiliations:** ^1^ Department of Clinical Genetics The 2nd Affiliated Hospital Nanjing Medical University, 262 North Zhongshan Road, Nanjing 210003, Jiangsu, China; ^2^ Center for kidney diseases The 2nd Affiliated Hospital Nanjing Medical University, 262 North Zhongshan Road, Nanjing 210003, Jiangsu, China; ^3^ School of Stomatology Xuzhou Medical University, No. 209 Tongshan Road, Xuzhou 221000, Jiangsu, China

## Abstract

Macrophage alternative activation is involved in kidney fibrosis. Previous researches have documented that the transcriptional regulators Yes-associated protein (Yap)/transcriptional coactivator with PDZ-binding motif (Taz) are linked to organ fibrosis. However, limited knowledge exists regarding the function and mechanisms of their downstream molecules in regulating macrophage activation and kidney fibrosis. In this paper, we observed that the Hippo pathway was suppressed in macrophages derived from fibrotic kidneys in mice. Knockout of Taz or Tead1 in macrophages inhibited the alternative activation of macrophages and reduced kidney fibrosis. Additionally, by using bone marrow–derived macrophages (BMDMs), we investigated that knockout of Taz or Tead1 in macrophages impeded both cell proliferation and migration. Moreover, deletion of Tead1 reduces p-Smad3 and Smad3 abundance in macrophages. And chromatin immunoprecipitation (ChIP) assays showed that Tead1 could directly bind to the promoter region of Smad3. Collectively, these results indicate that Tead1 knockout in macrophages could reduce TGF*β*1-induced phosphorylation Smad3 via transcriptional downregulation of Smad3, thus suppressing macrophage alternative activation and IRI-induced kidney fibrosis.

## 1. Introduction

Macrophages have a crucial function in the process of wound healing and regeneration as they connect the early stage of inflammation with the subsequent phases of tissue repair and regeneration. After renal injury, such as renal ischemia–reperfusion injury (IRI), the macrophage phenotype varies, ranging from the proinflammatory phenotype to the anti-inflammatory phenotype, contributing to tissue repair and fibrosis [1]. The presence of alternative macrophages in tissues for a prolonged period is recognized to prolong the damage phase, ultimately leading to ineffective kidney repair and contributing to the transition from AKI to CKD [2]. The molecular mechanisms that regulate alternative macrophage activation have become clearer over the past decade. During kidney fibrosis, however, the mechanisms regulating macrophage polarization still need further exploration.

The Hippo pathway is evolutionarily preserved and has been found to have a wider range of functions, such as maintaining tissue balance, promoting wound healing and regeneration, supporting immunity, and contributing to tumorigenesis [3]. The nuclear activity of Yap/Taz in Hippo signaling stimulates the progression of fibrotic cellular characteristics, including the differentiation of myofibroblasts and enhanced potential for matrix remodeling in liver [4], kidney [5], lung [6], and skin [7] fibroblasts. Many of these effects are mediated by transcriptional regulatory factors Yap and Taz, which regulate gene expression by controlling the transcription factor Tead family [8]. Continuous Taz activation promotes epithelial maladaptive repair, and it is likely that Hippo signaling contributes to kidney fibrosis [5, 9]. In our published research, we discovered that Yap/Taz mediates macrophage alternative activation by Wnt5a and promotes kidney fibrosis [10]. Nevertheless, the specific function and processes of Tead1 in controlling alternative macrophage activation and its impact on kidney fibrosis remain unclear.

The transition from macrophage to myofibroblast (MMT), which is an important step in the development of fibrosis, is induced by the canonical TGF*β*1–Smad3 signaling in macrophages [11]. Furthermore, TGF*β*1 induces macrophage polarization and differentiation and promotes myofibroblast accumulation [12]. After renal damage, the alternative activation is induced by short-term activation of TGF*β*1, leading to immunosuppression, matrix restructuring, and wound healing. Next, ongoing TGF*β*1 activation induces the MMT process [13]. The phosphorylation of the transcription factors Smad2 and Smad3 at their C-terminus is a crucial stage in the transition of macrophages induced by TGF*β*1 [14]. Knockout of Smad3 in macrophages leads to a decrease in myofibroblasts and diminishes fibrosis following UUO injury [15]. Yap/Taz have been identified as mechanoregulators of TGF*β*1 signaling and renal fibrogenesis. Pharmacological disruption of the Tead-Yap complex downregulates Yap and Taz expression, which is associated not only with reduced Smad3 nuclear accumulation but also with diminished Smad3 levels after TGF*β*1 stimulation [5]. Therefore, it is highly possible that Taz/Tead may be involved in the induction of alternative macrophage activation and contribute to kidney fibrosis via regulation of Smad3.

In our study, we discovered that conditional knockout of Taz or Tead1 in macrophages in kidney fibrosis mouse model resulted in a decrease in both kidney fibrosis and the alternative activation of macrophages. In vitro, specific ablation of Taz/Tead1 markedly inhibited cell macrophage proliferation and migration. Mechanistically, Tead1 as a primary downstream effector of Taz in Hippo signaling could promote TGF*β*1-induced macrophage alternative activation via transcriptional regulation of Smad3.

## 2. Materials and Methods

### 2.1. Mice and Animal Models

The male C57BL/6 mice, aged between 6 and 8 weeks and weighing between 18 and 20 g, were obtained from the Experimental Animal Center at Nanjing Medical University. The mice were kept in an environment with an appropriate temperature and humidity, following a light/dark cycle of 12 hr each. Every mouse was given unrestricted availability to both nourishment and water. To induce anesthesia in mice, pentobarbital sodium (45 mg/kg) was administered via intraperitoneal injection. The mouse's left renal pedicle was clamped for a duration of 30 min in the kidney IRI model. Mice were euthanized at specified time intervals after IRI, and their kidneys were collected for further analysis.

Mice with the macrophage-specific mouse Csf1r promoter (019098; FVB-Tg (Csf1r-Cre/Esr1 ^*∗*^)) controlling the tamoxifen-inducible MerCreMer fusion protein expression were obtained from The Jackson Laboratory (Bar Harbor, ME). These mice were originally on a FVB background but were backcrossed for eight generations with C57BL/6J mice to generate Csf1r-Cre transgenic mice on a C57BL/6J background. TEAD1^fl/fl^ mice were sourced from Cyagen (Suzhou, China).

Through the crossbreeding of Tead1 floxed mice and Csf1r-Cre/Esr1 ^*∗*^ transgenic mice, a mouse population exhibiting heterozygosity for the Tead1 floxed allele was produced, characterized by the genotype Csf1r-Cre^+/−^, Tead1^fl/wt^. Various offspring with different genotypes (Csf1r-Cre^−/−^, Tead1^fl/wt^ and Csf1r-Cre^−/−^, Tead1^fl/fl^; Csf1r-Cre^+/−^, Tead1^fl/fl^; and Csf1r-Cre^+/−^, Tead1^fl/wt^) were produced by breeding these mice with homozygous Tead1 floxed mice (genotype, Tead1^fl/fl^). The study utilized mice with the Csf1r-Cre^+/−^, Tead1^fl/fl^ genotype, as well as their gender-matched littermates with the Csf1r-Cre^−/−^, Tead1^fl/fl^ genotype. PCR assay was used to genotype the mouse tail by extracting DNA. Mice with Csf1r-Cre^+/−^ and Tead1^fl/fl^ genotypes, along with their control littermates, underwent IRI surgery. They were then administered tamoxifen (T5648, Sigma‒Aldrich) intraperitoneally at a dosage of 25 mg/kg for a continuous period of 7 days, starting on Day 7 following the IRI procedure. The previously described method [10] was used to generate Csf1r-Cre^+/−^ Taz^fl/fl^ mice through the breeding strategy.

All procedures conducted at Nanjing Medical University College were approved by the Ethics Committee and followed the ARRIVE Guidelines (Animal Research: Reporting of In Vivo Experiments) and the National Institutes of Health Guide for the Care and Use of Laboratory Animals to ensure ethical standards were met.

### 2.2. RNA Sequence

Briefly, RNA was prepared; library construction and sequencing were conducted on a BGISEQ-500 device at Beijing Genomics Institute (BGI). Gene expression levels were measured using RSEM. The NOISeq technique was used to identify genes with differential expression among the samples, and cluster was utilized for hierarchical clustering. GraphPad Prism8 was utilized to generate heatmaps. The expression pattern of the Hippo pathway in fibrotic kidneys after IRI was analyzed using gene set enrichment analysis (GSEA).

### 2.3. Cell Culture

BMDMs were obtained following previously described protocols [16]. Briefly, bone marrow cells were isolated and cultured in Dulbecco's Modified Eagle Medium (DMEM) supplemented with 10% fetal bovine serum (FBS), 10 ng/mL mouse macrophage colony-stimulating factor (M-CSF), and 1% antibiotics for 9 days. The culture medium was refreshed every other day. To generate BMDMs lacking Taz or Tead1, BMDMs from Csf1r-Cre^+/−^, Taz ^fl/fl^ or Csf1r-Cre^+/−^, Tead1 ^fl/fl^ mice were treated with 1 mM 4-OH tamoxifen at the beginning of the culture process. Control treatment was administered to bone marrow cells derived from Csf1r-Cre^−/−^, Tead1 ^fl/fl^ or Csf1r-Cre^−/−^, Taz ^fl/fl^ mice after exposure to 4-OH tamoxifen. On Day 9, BMDMs were cultured in serum-free medium and stimulated with TGF*β*1 (2 ng/mL) or treated with LPS (100 ng/*μ*L).

### 2.4. Macrophage Wound Healing and Migration Assay

BMDMs were cultured in a macrophage-inducing medium until they reached approximately 90% confluence. To create wound model, a sterile micropipette tip was used to scratch the cell monolayer. Additionally, a transwell system (Cat: PIEP12R48, Millipore) was utilized to evaluate cell migration.

### 2.5. Protein Extraction and Western Blot

BMDMs were lysed in 1 × SDS sample buffer, while kidneys were lysed with radioimmune precipitation assay buffer containing 0.1% SDS, 1% NP-40, 100 mg/mL PMSF, and 1% phosphatase I and II inhibitor cocktail and 1% protease inhibitor cocktail (Sigma-Aldrich) on ice. The lysates were then centrifuged at 13,000x*g* at 4°C for 30 min to collect the supernatants. The protein concentration in the supernatants was determined using the bicinchoninic acid protein assay (BCA Protein Assay Kit, Thermo Scientific) following the manufacturer's instructions. The primary antibodies used were as follows: anti-Smad3 (cat: ab40854, Abcam, 1 : 1,000), anti-p-Smad3 (cat: ab52903, Abcam, 1 : 1,000), anti-Tead1 (cat: ab106262, Abcam, 1 : 1,000), anti-Taz (cat: 83669, Cell Signaling Technology; 1 : 1,000), anti-p-Stat3 (Tyr-705) (cat:9145, Cell Signaling Technology, 1 : 1,000), anti-FN (cat: F3648, Sigma-Aldrich, 1 : 10,000), anti-*α*-SMA (cat: ab124964, Abcam, 1 : 10,000), anti-Arg-1 (cat:9819, Cell Signaling Technology, 1 : 1,000), anti-MR (cat: ab64693, 1 : 1,000), anti-Yap (cat: 4912S, Cell Signaling Technology, 1 : 1,000), anti-MST1 (cat: 14946, Cell Signaling Technology, 1 : 1,000), anti-MST2 (cat: 3952, Cell Signaling Technology, 1 : 1,000), anti-tubulin (cat: sc53646, Santa Cruz Biotechnology, 1 : 10,000), and anti-*β*-Actin (cat: sc47778, Santa Cruz Biotechnology; 1 : 1,000). The intensity of the signals was quantified using the National Institutes of Health ImageJ software package [17].

### 2.6. Real-Time qRT-PCR Assay

Trizol reagent (Invitrogen, catalog number 15596018) was used to extract total RNA from BMDMs, following the manufacturer's guidelines. The extracted RNA (1 *μ*g) was then used for cDNA synthesis using a cDNA synthesis kit (Vazyme, Cat. No. R223-01, 2020). PCR was performed on a Light Cycler 96 thermocycler (Roche) using the primer sequences listed in Table 1. To confirm the specificity of the PCR products, a melting curve analysis was conducted. The relative gene expression was calculated using the *ΔΔ*Ct method and normalized to the expression of *β*-actin.

### 2.7. Histology and Immunohistochemistry

Mouse kidney samples were fixed in 10% neutral formalin and subsequently embedded in paraffin. Thin sections with a thickness of three microns were stained using periodic acid–Schiff, Masson, and sirius red stains. The stained slides were observed under an OLYMPUS DP74 microscope that was equipped with a digital camera.

### 2.8. Immunofluorescence Staining

Samples of mouse kidneys were preserved by freezing kidney sections at a thickness of 3 *μ*m. To fix the sections, they were treated with 4% paraformaldehyde for 15 min. Following fixation, the sections were permeabilized with 0.2% Triton X-100 in 1x PBS for 5 min at room temperature. To block nonspecific binding, the sections were then incubated in 2% donkey serum for 60 min. Immunostaining was performed using various antibodies, including anti-F4/80 (cat: 14–4801, eBioscience, San Diego, CA, USA), anti-FN (cat: F3648, Sigma‒Aldrich), anti-MR (cat: ab64693, Abcam), anti-Ki67 (ab16667, Abcam), anti-*α*-SMA (cat: ab124964, Abcam), anti-p-Smad3 (cat: ab52903, Abcam), anti-Taz (cat: 83669, Cell Signaling Technology), and anti-Tead1 antibody (cat: ab106262, Abcam). To visualize the nuclei, the tissues were stained with DAPI. The slides were observed using an OLYMPUS DP74 and BX53 Epifluorescence microscope equipped with a digital camera. Confocal images were obtained using confocal microscopy (OLYMPUS, FV3000). To quantify the number of F4/80-positive macrophages, 10 randomly chosen fields within the cortical area were examined for each sample using a microscope at 400x magnification. The average number of positive cells per section was then calculated.

### 2.9. Kidney Monocyte/Macrophage Isolation

The kidney sections were frozen at a thickness suitable for further analysis. After cold perfusion with 1x PBS, the kidneys were extracted and fragmented. The fragments were then digested in DMEMs supplemented with 1 mg/mL collagenase and 0.1 mg/mL DNase for 1 hr at 37°C with periodic stirring. To obtain a suspension of individual cells, the fragments were passed through a 40-*μ*m mesh and filtered. To enrich macrophages from the single-cell suspension, CD115 microbeads and a MACS column were used following the manufacturer's instructions (Miltenyi Biotech, Bergisch-Gladbach, Germany).

### 2.10. ChIP Analysis

The ChIP experiments were conducted using a Thermo Pierce Magnetic ChIP Kit, in accordance with the guidelines provided by the manufacturer. To summarize, cells were fixed with 0.9% formaldehyde and quenched with glycine. Subsequently, an ultrasonic crushing apparatus was used to fragment the cells. Afterward, the liquid above was gathered and diluted, and the indicated antibodies were included for an overnight incubation period. The DNA–protein complexes were immunoprecipitated with ChIP-Grade Protein A/G Magnetic Beads and then underwent washing, elution, and reverse crosslinking procedures. Afterward, the DNA was purified and identified through agarose gel electrophoresis. The forward primer for the predicted promoter region of Smad3 is 5′GGCTAGCCTGATAGGGAGGCTGAAACAGGAT, and the reverse primer is 3′GGCTAGCCTGATAGGGAGGCTGAAACAGGAT.

### 2.11. Statistical Analyses

GraphPad Prism 8 software were used statistical analysis. The mean standard error (SE) was used to present the analyzed data. Group comparisons were conducted using one-way analysis of variance (ANOVA), followed by the Student–Newman–Keuls test. Additionally, unpaired *t*-tests were used to compare two groups. Statistical significance was defined as a *p* value below 0.05.

## 3. Results

### 3.1. The Hippo Signaling Pathway Is Suppressed in Fibrotic Kidney-Derived Macrophages

**Figure 1:**
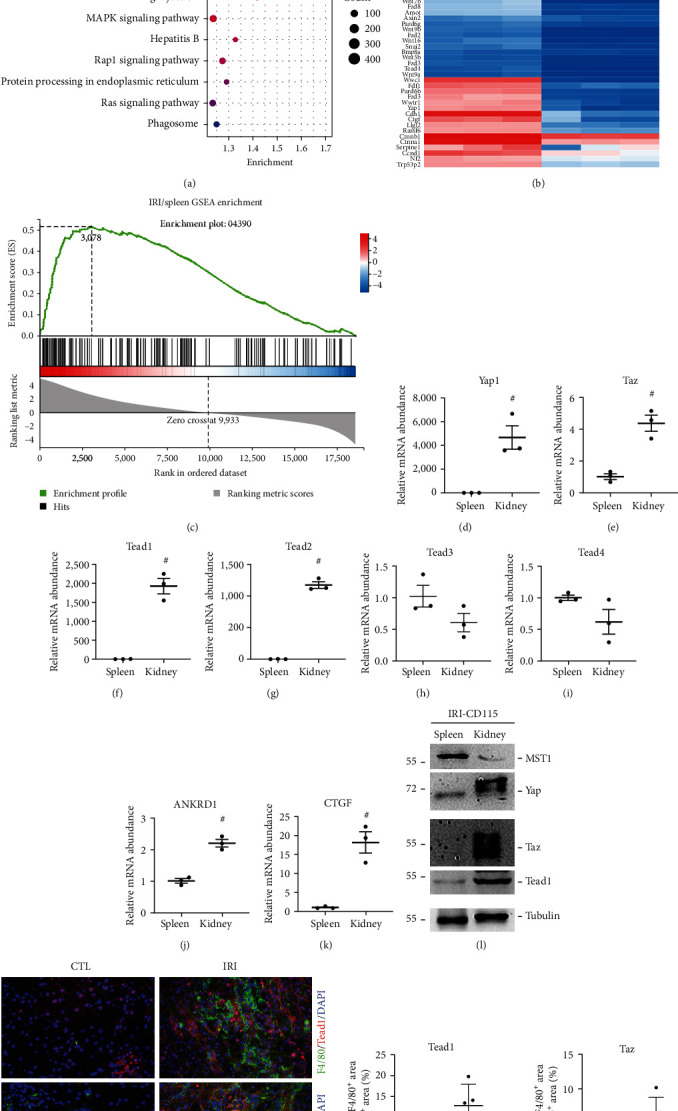
The Hippo signaling pathway is suppressed in fibrotic kidney-derived macrophages. (a) KEGG enrichment pathway bubble diagram of macrophages derived from the spleen and IRI (*n* = 3). (b) Heatmaps from RNA-seq analysis showing differentially expressed genes in CD115 magnetic bead-sorted monocytes/macrophages from fibrotic kidneys after IRI (*n* = 3). The expression of Yap, Taz, and Tead in the Hippo signaling pathway was significantly increased. (c) Gene set enrichment analysis (GSEA) for the Hippo signaling pathway in IRI model mice kidneys. (d–k) The mRNA abundance of Yap1 (d), Taz (e), Tead1-4 (f–i), ANKRD1 (j), and CTGF (k). (l) Western blotting showing the expression level of Mst1, Yap, Taz, and Tead1 in CD115 magnetic bead-sorted monocytes/macrophages from fibrotic kidneys after IRI. (m, n) Representative immunofluorescence images (left) and quantitative analysis (right) showing the induction of Tead1 or Taz in F4/80-positive macrophages within the fibrotic kidneys after IRI (*n* = 5; scale bar, 100 *μ*m). Each point represents the analysis from a randomly selected visual field within a biological sample. CTL, control; IRI, ischemia–reperfusion injury; ^#^*p* < 0.05. The data are presented as the means ± SDs.

Studies have shown that the Hippo signaling pathway plays a role in the progression of kidney fibrosis [9, 18, 19]. The investigation of Yap and Taz's involvement in fibrosis has prompted research into these factors [20]. However, the specific downstream mediator of the Hippo pathway in regulating macrophage activation and its contribution to kidney fibrosis is still not fully understood. CD115, also known as CSF1R, which is enriched in macrophages as a cell marker, has been widely used [21]. Therefore, we used IRI model of and employed CD115 magnetic beads to isolate monocytes/macrophages from the injured kidneys for subsequent RNA sequencing (RNA-seq) analysis. As there are fewer macrophages in the normal kidney, we used macrophages isolated from the spleen as controls. RNA-seq data showed that Hippo signaling pathway is enriched in KEGG analysis with *p* < 0.05 (Figure 1(a)). The expression of Hippo signaling pathway components, including Yap, Taz, and Tead, was significantly upregulated as shown in Figure 1(b). The enrichment of the Hippo pathway was additionally evaluated through GSEA. The abundance of the Hippo signaling protein was significantly increased in fibrotic kidneys following IRI, as shown in Figure 1(c). We also verified the RNA-seq data by qRT-PCR analysis (Figures 1(d), 1(e), 1(f), 1(g), 1(h), 1(i), 1(j), and 1(k)). MST1 as a core component of the Hippo pathway inhibits Yap and Taz nuclear accumulation and activity. And Mst1 was significantly decreased in IRI model of kidney fibrosis which indicated that Hippo signaling was suppressed after IRI in kidney (Figure 1(l)). In addition, immunofluorescence staining images showed that Tead1 and Taz were highly expressed in IRI kidneys compared to either the spleen or normal kidney group among the infiltrated F4/80-positive macrophages (Figures 1(m) and 1(n)). Therefore, we demonstrated that Hippo signaling was suppressed in fibrotic kidneys derived macrophages.

### 3.2. Knockout of Taz in Macrophages Attenuates IRI-Induced Kidney Fibrosis

**Figure 2:**
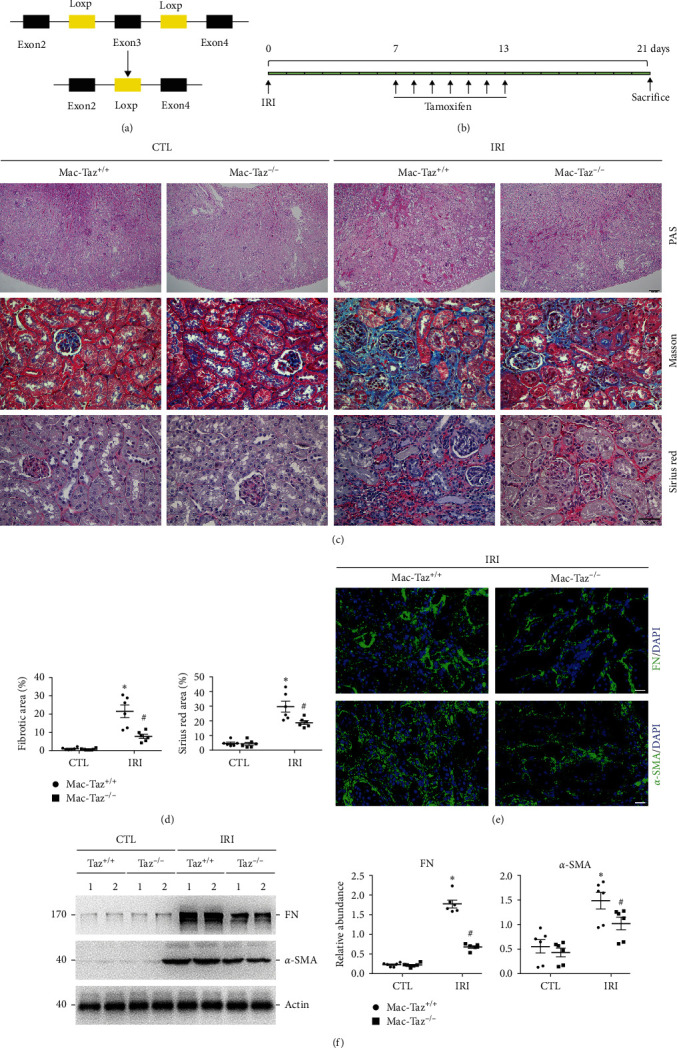
Knockout of Taz in macrophages attenuates IRI-induced kidney fibrosis. (a) Pattern diagram for generating Csf1r-Cre^+/−^ Taz^fl/fl^ mice. (b) Strategy for 4-hydroxytamoxifen injection and IRI surgery in mice. (c) Periodic acid–Schiff (PAS, 100x magnification), Masson's trichrome, and sirius red staining of kidney sections from the indicated groups (*n* = 6; scale bar, 100 *μ*m). (d) Quantification of the fibrotic area and the percentage of sirius red-positive area. Each point represents the analysis from a randomly selected visual field within a biological sample. (e) Representative immunofluorescence images for FN and *α*-SMA in IRI kidneys from the indicated groups (*n* = 6; scale bar, 50 *μ*m). (f) Western blot band (left) and quantitative analysis (right) of FN and *α*-SMA in IRI kidneys among the indicated groups (*n* = 4−6).  ^*∗*^*p* < 0.05 versus contralateral kidneys; ^#^*p* < 0.05 versus Mac-Taz^+/+^ fibrotic kidneys after IRI. The data are presented as the means ± SDs.

In our previously published study, we identified that knockout of Taz in macrophages led to reduce the activation of alternative macrophages and the development of kidney fibrosis in mice with UUO nephropathy [10]. To investigate the functions and underlying mechanisms of Taz induction in the IRI model of kidney fibrosis, we generated a mouse model using the Cre-LoxP system to specifically induce the knockout of Taz in macrophages (Figure 2(a)). Mac-Taz^−/−^ mice were created by injecting 4-hydroxytamoxifen intraperitoneally for 5 consecutive days into Csf1r-Cre^+^, Taz^fl/fl^ mice to induce macrophage ablation. Mac-Taz^+/+^ was the name given to the Csf1r-Cre^−^, Taz^fl/fl^ littermates after being injected with 4-hydroxytamoxifen (Figure 2(b)). Kidney histology of conditional knockouts of macrophage Taz was comparable with that of controls. Nevertheless, the interstitial fibrotic area and overall collagen content in the IRI kidneys of the knockouts were significantly reduced compared to their control littermates (Figures 2(c) and 2(d)). Furthermore, the immunofluorescence staining images and western blot assays revealed a significant reduction in the levels of FN and *α*-SMA in the IRI kidneys of Mac-Taz^−/−^ compared to Mac-Taz^+/+^ kidneys (Figures 2(e) and 2(f)). Therefore, the findings suggested that knockout of Taz in macrophages reduces kidney fibrosis caused by IRI.

### 3.3. Knockout of Taz in Macrophages Reduces Macrophage Infiltration and Alternative Activation in IRI-Induced Kidney Fibrosis

**Figure 3:**
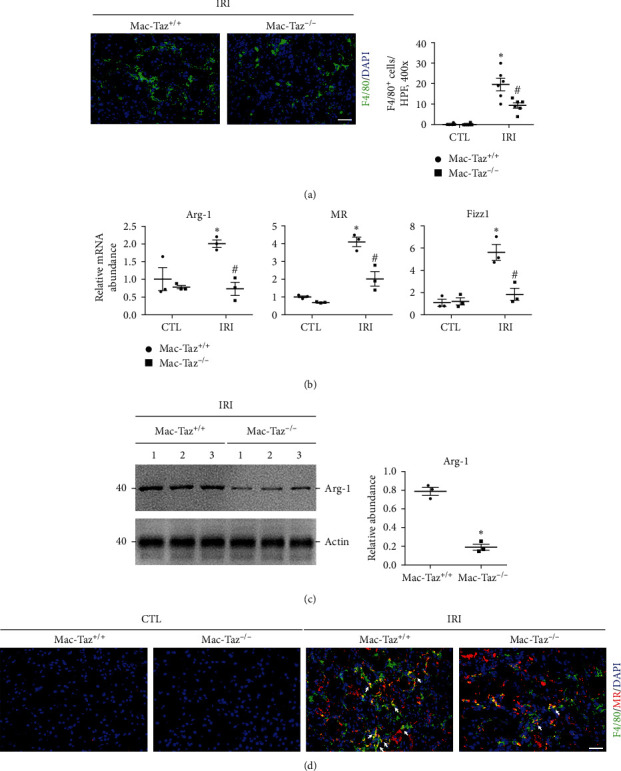
Knockout of Taz in macrophages reduces macrophage infiltration and alternative activation in IRI-induced kidney fibrosis. (a) Representative micrographs of immunofluorescence images for F4/80-positive cells in IRI kidneys among the indicated groups (*n* = 6; scale bar, 50 *μ*m). Each point represents the analysis from a randomly selected visual field within a biological sample. (b) The mRNA abundance of Arg-1, MR, and Fizz1 in macrophages from contralateral and IRI kidneys at Day 21 after surgery. (c) Western blot assay (left) and quantitative analysis (right) of Arg-1 in IRI kidneys among the indicated groups. (d) Representative immunostaining images for F4/80 and MR in IRI kidneys among the indicated groups. White arrows indicate both F4/80- and MR-positive cells. Scale bar, 50 *μ*m.  ^*∗*^*p* < 0.05 versus Mac-Taz^+/+^ contralateral kidneys; ^#^*p* < 0.05 versus Mac-Taz^+/+^ fibrotic kidneys after IRI. The data are presented as the means ± SDs.

Long-lasting M2 macrophages trigger the MMT process as a key checkpoint for kidney fibrosis [13]. Knockout of Taz in macrophages reduced the development of kidney fibrosis caused by IRI. Next, we continued to investigate if knockout of Taz hinders the accumulation of macrophages and alternative activation in IRI kidneys. In Figure 3(a), there was a significant reduction in the number of F4/80-positive macrophages observed in the Mac-Taz^−/−^ kidneys when compared to Mac-Taz^+/+^ kidneys following IRI. After IRI, the macrophages from Mac-Taz^+/+^ kidneys exhibited significant upregulation in the mRNA expression of Arg-1, Fizz1, and MR, whereas Mac-Taz^−/−^ kidneys showed considerably lower levels of expression in macrophages (Figure 3(b)).CD115 microbeads were used to sort macrophages from fibrotic kidneys, and western blot analysis demonstrated a notable reduction in the levels of Arg-1 within macrophages derived from IRI kidneys of Mac-Taz^−/−^ mice as compared to macrophages from Mac-Taz^+/+^ mice (Figure 3(c)). Moreover, immunofluorescence staining showed that Taz knockout in macrophages significantly decreased the number of F4/80-positive macrophages but was concomitant with low expression of MR, a macrophage alternative activation marker (Figure 3(d)). Hence, these findings suggest that knockout of Taz in macrophages reduces the accumulation of macrophages, alternative activation, and kidney fibrosis following IRI in mice.

### 3.4. Knockout of Taz in Macrophages Inhibits Macrophage Proliferation and Migration in BMDMs

**Figure 4:**
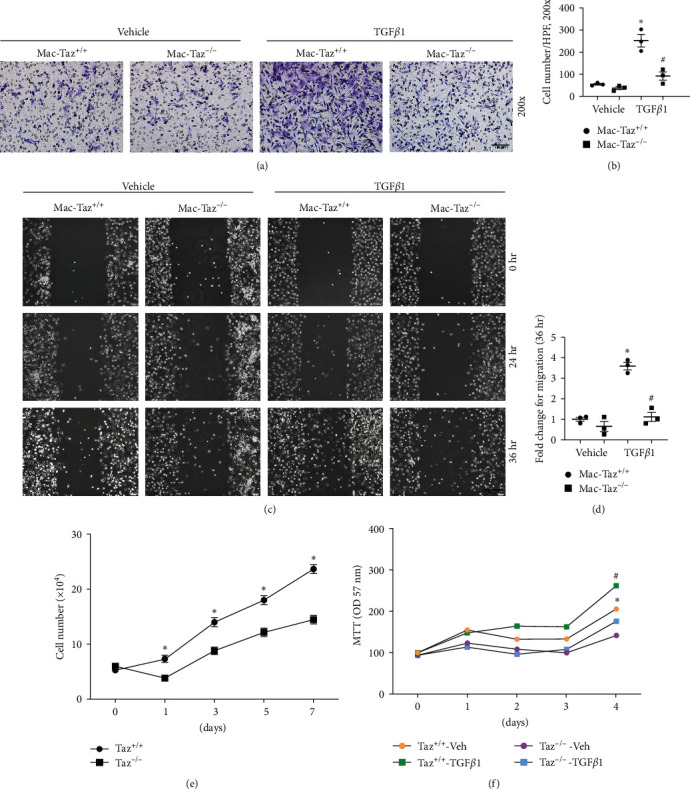
Knockout of Taz in macrophages inhibits macrophage proliferation and migration in BMDMs (a, b). Representative images (a) and quantitative analysis (b) of transwell migration assay in cultured BMDMs treated with or without TGF*β*1 (2 ng/mL) for 24 hr among Taz^+/+^ and Taz^−/−^ groups as indicated (*n* = 3; scale bar, 100 *μ*m). (c, d) Representative images (c) and quantitative analysis (d) of the wound healing test in cultured BMDMs from different groups as indicated (*n* = 3; scale bar, 100 *μ*m). Each point represents the analysis from the average of three randomly selected visual fields within a biological sample. (e) Cell counting assay in cultured BMDMs among Taz^+/+^ and Taz^−/−^ groups as indicated. (f) MTT proliferation assay in cultured BMDMs treated with or without TGF*β*1 among the indicated groups.  ^*∗*^*p* < 0.05 versus cultured Taz^+/+^ BMDMs treated with vehicle; ^#^*p* < 0.05 versus cultured Taz^+/+^ BMDMs treated with TGF*β*1. The data are presented as the means ± SDs.

After confirming that Taz may induce alternative macrophage activation in IRI kidneys, we then continued to explore whether Taz could regulate macrophage proliferation and migration. BMDMs obtained from mice with the Csf1r-Cre^+^ genotype and Taz^fl/fl^ genotype were subjected to a 5-day treatment with 4-hydroxytamoxifen to induce knockout of the Taz gene. Subsequently, they were stimulated with TGF*β*1 for 24 hr to induce an alternative activation of macrophages. Transwell migration assay results indicated that TGF*β*1 significantly increased the migration of macrophages, whereas the Mac-Taz^−/−^ group exhibited considerably lower macrophage migration compared to the Mac-Taz^+/+^ group (Figures 4(a) and 4(b)). Wound healing tests also showed that macrophages with Taz possessed stronger migration capabilities than macrophages in the Taz gene knockout group (Figures 4(c) and 4(d)). For proliferation capability, we first counted the cell number in cultured BMDMs and found that knockout of Taz impaired macrophage proliferation capability (Figure 4(e)). Furthermore, the MTT assay demonstrated that the Mac-Taz^−/−^ groups were less proliferative than the Mac-Taz^+/+^ groups in TGF*β*1-treated macrophages (Figure 4(f)). Therefore, these findings suggest that knockout of Taz in macrophages inhibits macrophage proliferation and migration.

### 3.5. Taz/Tead1 Mediates TGF*β*1-Induced Macrophage Alternative Activation

**Figure 5:**
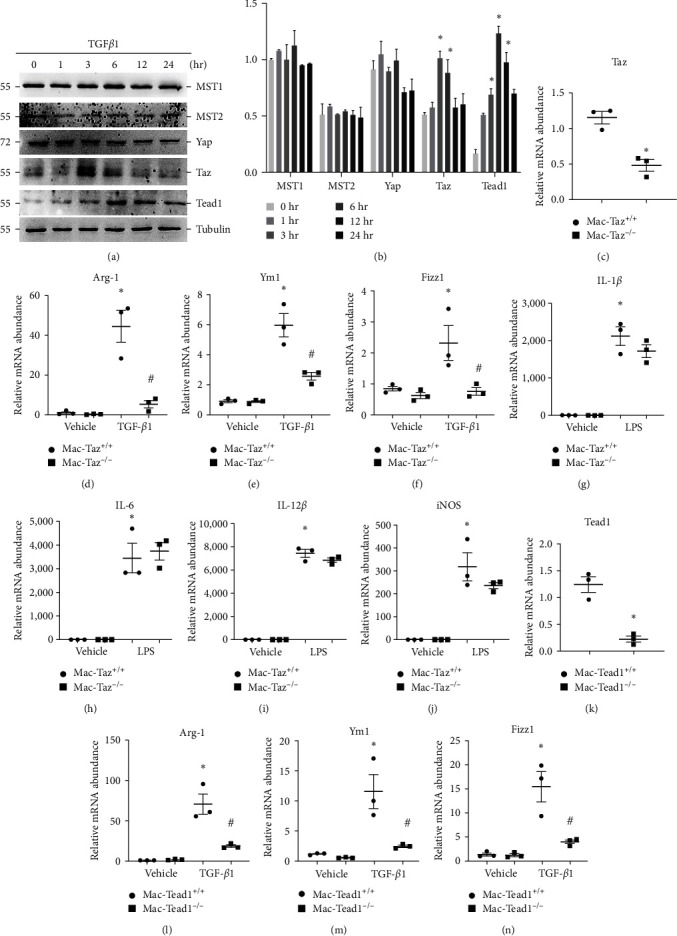
Taz/Tead1 mediates TGF*β*1-induced macrophage alternative activation. (a, b) The protein expression level of MST1, MST2, Yap, Taz, and Tead1 in BMDMs that were treated with TGF*β*1 (2 ng/mL) for different durations as indicated. (c) Verification of 4-hydroxytamoxifen-induced Taz knockout in cultured BMDMs by qRT-PCR analysis. (d–j) qRT-PCR analysis of Arg-1 (d), Ym1 (e), and Fizz1 (f) in cultured BMDMs treated with or without TGF*β*1 (2 ng/mL) for 24 hr among the indicated Taz^+/+^ and Taz^−/−^ groups. (g–j) qRT-PCR analysis of IL-1*β* (g), IL-6 (h), IL-12*β* (i), and iNOS (j) in cultured BMDMs treated with or without LPS (1 *μ*g/mL) among the indicated groups. (k) Verification of 4-hydroxytamoxifen-induced Tead1 knockout in cultured BMDMs by qRT-PCR analysis. (l–n) qRT‒PCR analysis of Arg-1 (l), Ym1 (m), and Fizz1 (n) in cultured BMDMs treated with or without TGF*β*1 (2 ng/mL) for 24 hr among the Tead1^+/+^ and Tead1^−/−^ groups as indicated.  ^*∗*^*p* < 0.05 versus cultured Taz^+/+^ BMDMs treated with vehicle; ^#^*p* < 0.05 versus cultured Taz^+/+^ BMDMs treated with TGF*β*1. The data are presented as the means ± SDs.

We then wanted to specifically clarify the processes through which Taz mediates TGF*β*1-stimulated macrophage alternative activation. Based on the RNA-seq results from CD115-sorted macrophages in IRI kidneys (Figure 1), the downstream transcription factor is likely to be Tead1 or Tead2. Western blot results showed that Taz was markedly increased during TGF*β*1 stimulation which is followed by an increase in Tead1 expression (Figures 5(a) and 5(b)). These results indicated that Tead1 may be a downstream effector of Taz. We first verified the knockout of Taz in cultured BMDMs (Figure 5(c)). Then, we found that TGF*β*1 treatment for 24 hr could significantly induce the expression of Arg-1, Ym-1, and Fizz1 but much less in Mac-Taz^−/−^ macrophages than in Mac-Taz^+/+^ macrophages (Figures 5(d), 5(e), and 5(f)). Moreover, we investigated whether Taz is involved in macrophage M1 polarization. However, qPCR results illustrated that LPS treatment for 24 hr induced high expression of IL-1*β*, IL-6, IL-12*β*, and iNOS, but no significant difference was found between Mac-Taz^−/−^ and Mac-Taz^+/+^ macrophages (Figures 5(g), 5(h), 5(i), and 5(j)). Additionally, we found that TGF*β*1 treatment for 12 and 48 hr could also significantly induce the expression of Arg-1, Ym-1, and Fizz1 but to a much lesser extent in TAZ-deficient macrophages compared to Taz^+/+^ macrophages (Figure S2). Moreover, qPCR results illustrated that LPS treatment for 12 hr could also induce high expression of IL-1*β*, IL-6, and iNOS, but no significant differences were found between TAZ-deficient and Taz^+/+^ macrophages (Figure S2).

Subsequently, we created a mouse model by employing a Cre-LoxP system to induce knockout of Tead1 in macrophages. BMDMs obtained from mice with Csf1r-Cre^+^ and Tead1^fl/fl^ genotypes were exposed to 4-hydroxytamoxifen for a duration of 5 days in order to induce knockout of the Tead1 gene. Subsequently, these BMDMs were stimulated with TGF*β*1 for a period of 24 hr to induce an alternative activation of macrophages. Knockout of Tead1 was verified in cultured BMDMs (Figure 5(k)). The expression levels of Arg-1, Ym-1, and Fizz1 were much lower in the Mac-Tead1^−/−^ group than in the Mac-Tead1^+/+^ group (Figures 5(l), 5(m), and 5(n)). These results indicated that Tead1, as a downstream effector of Taz, mediates TGF*β*1-induced macrophage alternative activation.

### 3.6. Knockout of Tead1 in Macrophages Inhibits Macrophage Proliferation and Migration

**Figure 6:**
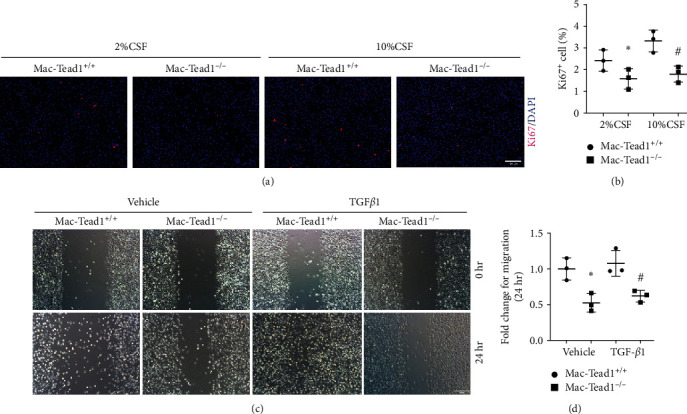
Knockout of Tead1 in macrophages inhibits macrophage proliferation and migration. (a, b) Ki67 immunofluorescence staining (a) and quantification (b) of Ki67-positive BMDMs treated with or without TGF*β*1 (2 ng/mL) for 24 hr among the Tead1^+/+^ and Tead1^−/−^ groups as indicated (scale bar, 100 *μ*m). (c, d) Representative images (c) and quantitative analysis (d) of the wound healing test in cultured BMDMs from different groups as indicated (*n* = 3; scale bar, 100 *μ*m). Each point represents the analysis from the average of three randomly selected visual fields within a biological sample.  ^*∗*^*p* < 0.05 versus cultured Tead1^+/+^ BMDMs treated with vehicle; ^#^*p* < 0.05 versus cultured Tead1^+/+^ BMDMs treated with TGF*β*1. The data are presented as the means ± SDs.

Based on the above results, we continued to test whether Tead1 could regulate macrophage proliferation and migration as well. Immunofluorescence staining for Ki67 showed that macrophages with Tead1 possessed stronger proliferative capabilities than those with Tead1 gene knockout (Figures 6(a) and 6(b)). In addition, the wound healing test showed that macrophages with Tead1 possessed stronger migration capabilities compared with the Tead1 gene knockout group (Figures 6(c) and 6(d)).

**Figure 7:**
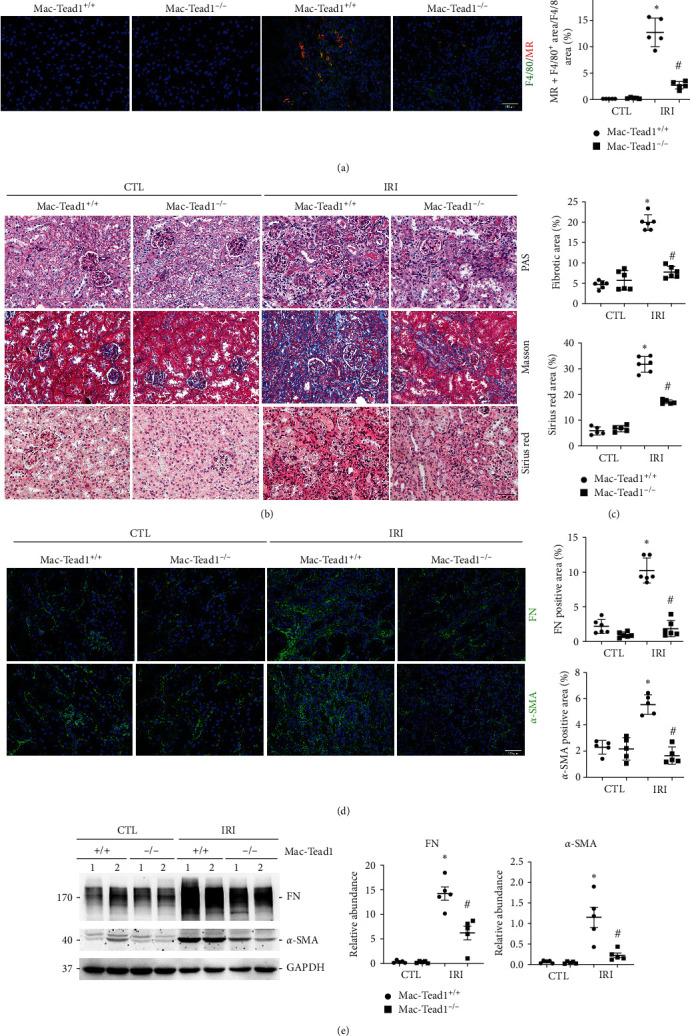
Knockout of Tead1 in macrophages attenuates IRI-induced kidney fibrosis. (a) Representative confocal immunofluorescence images and quantitative analysis (right) of F4/80 and MR in IRI kidneys among the indicated groups (*n* = 5). Scale bar, 100 *μ*m. (b) Periodic acid–Schiff (PAS), Masson's trichrome, and sirius red staining of kidney sections from the indicated groups (*n* = 6; scale bar, 100 *μ*m). (c) Quantification of the fibrotic area and the percentage of sirius red-positive area. (d) Representative immunofluorescence images (left) and quantitative analysis (right) of FN and *α*-SMA in IRI kidneys from the indicated groups (*n* = 5−6; scale bar, 100 *μ*m). Each point represents the analysis from a randomly selected visual field within a biological sample. (e) Western blot assay (left) and quantitative analysis (right) of FN and *α*-SMA in IRI kidneys among the indicated groups.  ^*∗*^*p* < 0.05 versus contralateral kidneys; ^#^*p* < 0.05 versus Mac-Tead1^+/+^ fibrotic kidneys after IRI. The data are presented as the means ± SDs.

The results above indicated that Tead1 regulates macrophage proliferation and migration. We observed a certain level of Tead1 expression in macrophages in the IRI kidney in single-cell RNA sequencing public data (Figure S1) [22]. Thus, we assumed that knockout of Tead1 in macrophages attenuates IRI nephropathy. Unsurprisingly, immunofluorescence staining showed that the number of F4/80-positive macrophages was significantly increased in IRI kidneys, which was much lower in the Mac-Tead1^−/−^ group with concomitant low MR expression than in the Mac-Tead1^+/+^ group (Figure 7(a)). The kidney histology of mice with conditional knockout of macrophage Tead1 was comparable to that of controls. However, the Mac-Tead1^−/−^ group had significantly reduced interstitial fibrotic area and total collagen content compared to the Mac-Tead1^+/+^ group (Figures 7(b) and 7(c)). Furthermore, the immunofluorescence images and western blotting analyses revealed a significant reduction in the levels of FN and *α*-SMA in the IRI kidneys of Mac-Tead1^−/−^ compared to Mac-Tead1^+/+^ kidneys (Figures 7(d) and 7(e)). Therefore, the findings suggested that knockout of Tead1 in macrophages reduces the progression of kidney fibrosis caused by IRI.

### 3.7. Knockout of Tead1 Reduces p-Smad3/Smad3 Abundance in Macrophages

**Figure 8:**
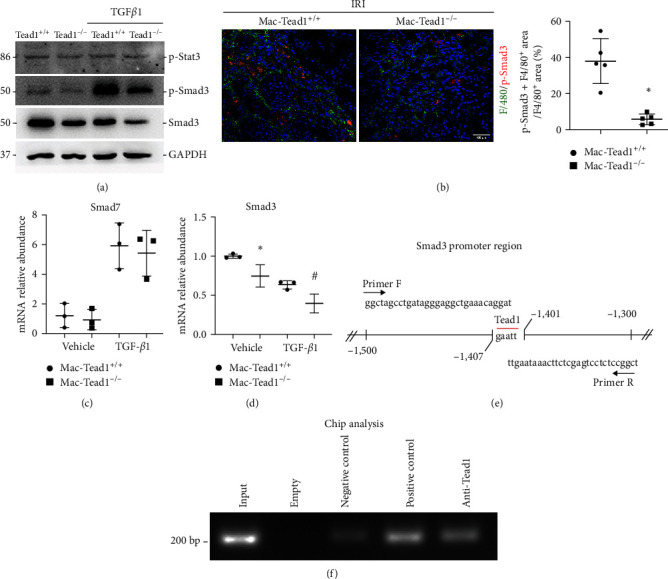
Knockout of Tead1 reduces p-Smad3/Smad3 abundance in macrophages. (a) Western blot assay for p-Stat3, p-Smad3, and Smad3 expression in BMDMs treated with or without TGF*β*1 (2 ng/mL) for 24 hr among the Tead1^+/+^ and Tead1^−/−^ groups as indicated. (b) Representative confocal immunofluorescence images (left) and quantitative analysis (right) of F4/80 and p-Smad3 in IRI kidneys among the indicated groups (*n* = 5, scale bar, 100 *μ*m). Each point represents the analysis from a randomly selected visual field within a biological sample. (d, e) qRT-PCR analysis of Smad7 (c) and Smad3 (d) in cultured BMDMs treated with or without TGF*β*1 (2 ng/mL) for 24 hr among the Tead1^+/+^ and Tead1^−/−^ groups as indicated. (e) Predicted Tead1-binding sites in the Smad3 promoter region. (f) ChIP analysis showing that Tead1 binds to the Smad3 promoter region.  ^*∗*^*p* < 0.05 versus cultured Tead1^+/+^ BMDMs treated with vehicle; ^#^*p* < 0.05 versus cultured Tead1^+/+^ BMDMs treated with TGF*β*1. The data are presented as the means ± SDs.

Overall, the above results suggest that Taz/Tead1 regulates alternative macrophage activation. As it has been reported that alternative activation is associated with Jak3–Stat3 activation [23] and TGF*β*1–Smad3 signaling [14], we further investigated whether Tead1 regulates Stat3 and Smad3 activation. By using tamoxifen-induced Tead1 knockout BMDMs, we found that knockout of Tead1 had no effect on Stat3 phosphorylation but significantly decreased the abundance of Smad3 and p-Smad3 with or without TGF*β*1 treatment (Figure 8(a)). Moreover, immunofluorescence staining demonstrated that F4/80 and p-Smad3 were significantly decreased in the Mac-Tead1^−/−^ group (Figure 8(b)). These results indicated that the decrease in p-Smad3 abundance after knockout of Tead1 is primarily due to an overall decrease in total Smad3 expression and Tead1 may regulate Smad3 transcription.

Based on the above results, we further detected the RNA abundance of Smad3 in tamoxifen-induced Tead1 knockout BMDMs. The results showed that Tead1 knockout could markedly downregulate the expression of Smad3 with or without TGF*β*1 treatment (Figure 8(d)). Furthermore, there have been reports indicating that Yap/Taz impaired the phosphorylation of Smad3 and its transcriptional activity induced by TGF-*β*1 in human skin dermal fibroblasts and highlight the induction of Smad7 expression as a contributing factor [24]. Additionally, a direct interaction between Taz and Smad7 has been observed in myogenic cells [25]. Thus, we also detected the expression of Smad7 in Tead1 knockout BMDMs, but no difference was found Figure 8(c).

In order to ascertain if the impact of Tead1 knockout on Smad3 downregulation was a direct one, we predicted Tead1-binding sites in the Smad3 promoter region by UCSC and designed a pair of primers to amplify specific sequences in upstream and downstream regions of Tead1-binding sites. (Figure 8(e)). By using ChIP analysis, we demonstrated that Tead1 could directly bind to the promoter region of Smad3 (Figure 8(f)). Thus, these results indicated that Tead1 knockout could transcriptionally inhibit Smad3 expression in macrophages. Together, our findings suggested that Tead1 knockout in macrophages could reduce TGF*β*1-induced phosphorylation Smad3 via transcriptional downregulation of Smad3, thus suppressing macrophage alternative activation and kidney fibrosis caused by IRI.

## 4. Discussion

In our published research, we found that Yap/Taz medicates alternative activation of macrophages and promoting kidney fibrosis in UUO mice models [10]. Nevertheless, the specific mechanisms of the downstream effector of the Hippo pathway remain unclear. In this study, we found that knockout of Taz/Tead1 in macrophages reduced kidney fibrosis and macrophage alternative activation. And specific ablation of Taz/Tead1 markedly inhibited cell macrophage proliferation and migration. Mechanically, Tead1 knockout in macrophages could reduce TGF*β*1-induced phosphorylation Smad3 via transcriptional downregulation of Smad3.

Hippo signaling pathway has been proved to be associated with many fibrosis diseases [26]. Under physiological conditions, Lats1/2 complex is phosphorylated by sterile 20-like protein kinase (Mst1/2) formed complexes. These complexes then phosphorylate Yap and Taz, leading to their retention in the cytoplasm and subsequent degradation through proteasomes. Under pathological conditions, dephosphorylation of Lats1/2 complex drives nuclear translocation of Yap and Taz, where they can interact with Tead and regulate cell proliferation, survival, and differentiation [27, 28]. Likewise, in vivo, we found that Mst1 was significantly decreased, while Yap, Taz, and Tead1 and the expression of ANKRD1 and CTGF were significantly upregulated in IRI fibrotic kidneys. In vitro, we found that in TGF*β*1 stimulation time course, Taz was markedly increased followed by an increase in Tead1 expression. The late Tead1 expression that follows the transient change in Taz expression suggests a correlation between the Taz/Tead1 response. Taz is known to bind to Tead1 and enhance its transcriptional activity. Therefore, the increase in Tead1 expression may be a compensatory response to the initial change in Taz expression, ensuring that there is sufficient Tead1 available for interaction with Taz [29]. This correlation suggests that Taz and Tead1 may function together in a regulatory pathway, and their expression levels are tightly regulated to maintain proper cellular function. Moreover, we have shown that knockout of Taz or Tead1 in macrophages reduces the severity of IRI nephropathy in mice. Thus, we suggest that Tead1 as a primary downstream effector of Taz in Hippo signaling may become effective therapeutic targets for the treatment of chronic kidney disease.

An essential role of Tead1 is to regulate the transcriptional output of the Hippo signaling pathway. Tead1 engages in a complex with Yap/Taz, and together, they bind to a consensus DNA sequence 5′-CATTCC-3′, contributing to various biological processes [29, 30]. In this study, RNA-seq data showed that among Tead1–4, Tead1 and Tead2 were significantly elevated in macrophages from IRI kidneys. TEADs play a key role in heart morphogenesis, where Tead1 is essential for cardiomyocyte proliferation [31, 32]. Utilizing macrophage-specific Tead1 and Taz conditional knockout mice, we investigated the function of Taz/Tead1 in macrophages. In cultured BMDMs, we found that knockout of Taz or Tead1 could significantly inhibit macrophage proliferation and migration. In addition, by detecting macrophage polarization markers, we found that Taz/Tead1 was not involved in macrophage M1 polarization but mediated TGF*β*1-induced alternative activation.

Numerous cytokines and transcription factors influence alternative (M2) macrophage activation [33, 34, 35, 36]. According to our published research, alternative macrophage activation could be modulated by Wnt/*β*-catenin signaling via Stat3 activation [37]. Yap/Taz-mediated alternative macrophage activation resulted in Wnt5a-exacerbated kidney fibrosis [10]. In addition, Stat3 [38, 39] and TGF*β*1–Smad3 signaling [40] are two major pathways that mediate alternative macrophage activation. Here, in BMDMs, we found that Tead1 had no effect on Stat3 phosphorylation but significantly downregulated the expression level of p-Smad3 and total Smad3 regardless of TGF*β*1 stimulation. Moreover, knockout of Tead1 significantly downregulated p-Smad3 and Smad3 expression in vitro and in vivo. The findings were in line with prior research indicating that blockade of the TGF-*β*1-Smad3 signaling pathway could modulate alternative macrophage polarization and ameliorate IRI-induced renal fibrosis [15, 41].

We also noticed that Smad3 mRNA and protein levels decreased significantly in BMDMs incubated with TGF-*β*1. Indeed, this latter result is consistent with earlier findings showing that Smad3 expression was shown to be downregulated in UUO-induced fibrosis. The increase of phosphorylation Smad3 was accompanied by the decrease of total Smad3 following TGF-*β*1 treatment. Thus, Smad3 downregulation could represent a negative feedback loop controlling TGF-*β*1 responses, which is a cell type-specific event, occurring in fibroblastic cells [42]. In this study, we demonstrated that in macrophages, Tead1 knockout could further downregulate total Smad3 levels following TGF-*β*1 treatment. The downregulation of total Smad3 expression led to a decrease of TGF*β*1-induced phosphorylation Smad3, which modulated alternative macrophage polarization in Tead1^−/−^ group.

The crosstalk between TGF-*β*1 and Hippo signaling was early reported in human embryonic stem cells that knockout of Taz hindered accumulation of Smad2/3–4 complexes in the nucleus which results in inhibition of TGF*β*1 signaling [43]. Furthermore, it has been shown that Taz controls the TGF-*β*/Smad3 pathway by promoting the expression of Smad7 [24]. However, we found that knockout of Tead1 has no effect on the expression of Smad7. These results indicate that the regulation of Tead1 for Smad3 is not consistent with Taz during IRI-induced kidney fibrosis. By using ChIP analysis, we found that Tead1 could directly bind to the promoter region of Smad3. It suggested that Tead1 promotes alternative macrophage activation in kidney fibrosis by regulating Smad3 transcriptionally.

## 5. Conclusions

Ultimately, our study showed that Taz/Tead1 promotes alternative macrophage activation and drives the progression of kidney fibrosis. Tead1 knockout in macrophages could reduce TGF*β*1-induced phosphorylation Smad3 via transcriptional downregulation of Smad3. Targeting Tead1 in macrophages may become effective therapeutic targets for the treatment of chronic kidney disease.

## Figures and Tables

**Table 1:** Quantitative RT-PCR primers.

Gene	Reverse primers	Forward primers
*β*-Actin	TGGAATCCTGTGTGGCATCCATGAAA	TAAAACGCAGCTCAGTAACAGTCCG
MR	CCTTTCAGTCCTTTGCAAGC	CAAGGAAGGTTGGCATTTGT
Fizz1	CTGGATTGGCAAGAAGTTCC	CCCTTCTCATCTGCATCTCC
Ym1	TTTCTCCAGTGTAGCCATCCTT	TCTGGGTACAAGATCCCTGAA
Yap	TGTGCTGGGATTGATATTCCGTA	ACCCTCGTTTTGCCATGAAC
Taz	GTCGGTCACGTCATAGGACTG	CATGGCGGAAAAAGATCCTCC
Tead1	CCACACGGCGGATAGATAGC	GAGCGACTCGGCAGATAAGC
Tead2	CCACACTCTCTAGGGGTGGT	CAGACGCAGTTGACTCGTTC
Tead3	CTGAAAGCTCTGCTCGATGTC	CAACCAGCACAATAGCGTCCA
Tead4	TCCTCCGTCAGGATAATTTTGC	ACAATGATGCAGAGGGTGTATG
ANKRD1	TGGCACTGATTTTGGCTCCT	CTTGAATCCACAGCCATCCA
CTGF	CGGCTCTAATCATAGTTGGGTCT	AATGCTGCGAGGAGTGGGT
Smad3	GGCAGTAGATAACGTGAGGGA	CACGCAGAACGTGAACACC
Smad7	TTGGGTATCTGGAGTAAGGAGG	GGCCGGATCTCAGGCATTC
IL-1*β*	TGATGTGCTGCTGCGAGATT	TGCCACCTTTTGACAGTGATG
IL-6	CTGCAAGAGACTTCCATCCAG	AGTGGTATAGACAGGTCTGTTGG
IL-12*β*	AGTCCCTTTGGTCCAGTGTG	AGCAGTAGCAGTTCCCCTGA
iNOS	TCTATACCACTTCACAAGTCGGA	GAATTGCCATTGCACAACTCTTT

## Data Availability

The datasets used and/or analyzed during the current study are available from the corresponding author on reasonable request.

## Abbreviations

Arg-1: Arginase-1
*α*-SMA: *α*-Smooth muscle actin
BMM: Bone marrow–derived macrophage
ChIP: Chromatin immunoprecipitation
Fizz1: Found in inflammatory zone 1
FN: Fibronectin
GSEA: Gene set enrichment analysis
IRI: Ischemic–reperfusion injury
MR: Mannose receptor C type 1
MMT: Macrophage-to-myofibroblast transition
p-Stat: Phosphorylated Stat
qRT-PCR: Quantitative RT-PCR
RNA-seq: RNA sequencing
TGF*β*1: Transforming growth factor*β*1
Tead, Stat: Signal transducer and activator of transcription
MST1/2: Sterile 20-like protein kinase
TEA: Domain family member
Taz: Transcriptional coactivator with PDZ-binding motif
UUO: Unilateral ureter obstruction
Yap: Yes-associated protein.

## Supplementary Materials

Supplementary Materials The supplementary materials include supplementary Figures 1 and 2 and recording figure legends.
