# Supplementary material for: Taz/Tead1 Promotes Alternative Macrophage Activation and Kidney Fibrosis via Transcriptional Upregulation of Smad3
Source: J Immunol Res. 2024 Jul 30;2024:9512251. doi: 10.1155/2024/9512251 (PMC11303051; doi:10.1155/2024/9512251)

Supplementary Fig1 Tead1 expression in the IRI kidney in single-cell RNA sequencing public data.

1. Cell type identification projected on UMAP (b) Tead1 expression in single-cell RNA sequencing public data[1].

Supplementary Fig2. Taz mediates macrophage alternative activation. (a)Verification of siTaz influence in macrophages by qRT‒PCR analysis. (b-d) qRT‒PCR analysis of Arg-1 (b), Ym1 (c) and Fizz1 (d) in cultured macrophages treated with or without TGFβ1 (2 ng/ml) for 12h among the indicated groups. (e-g) qRT‒PCR analysis of IL-1β (e), IL-6 (f) and iNOS (g) in cultured macrophages treated with or without LPS (1 μg/ml) for 12h among the indicated groups. (h-j) qRT‒PCR analysis of Arg-1 (h), Ym1 (i) and Fizz1 (j) in cultured macrophages treated with or without TGFβ1 (2 ng/ml) for 48h indicated groups. *p<0.05 versus cultured Taz+/+ macrophages treated with vehicle; #p<0.05 versus cultured Taz+/+ macrophages treated with TGFβ1or LPS. The data are presented as the means ± SDs.

Supplementary Fig1


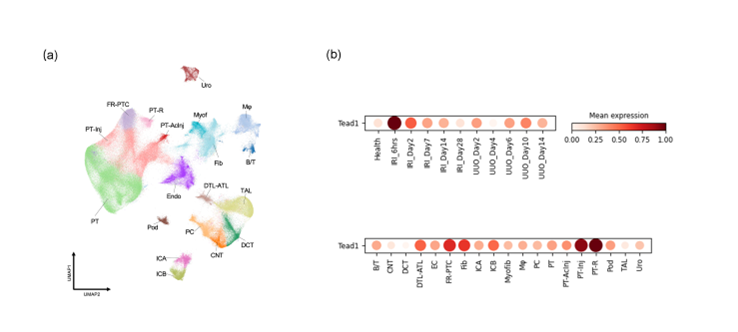


Supplementary Fig2


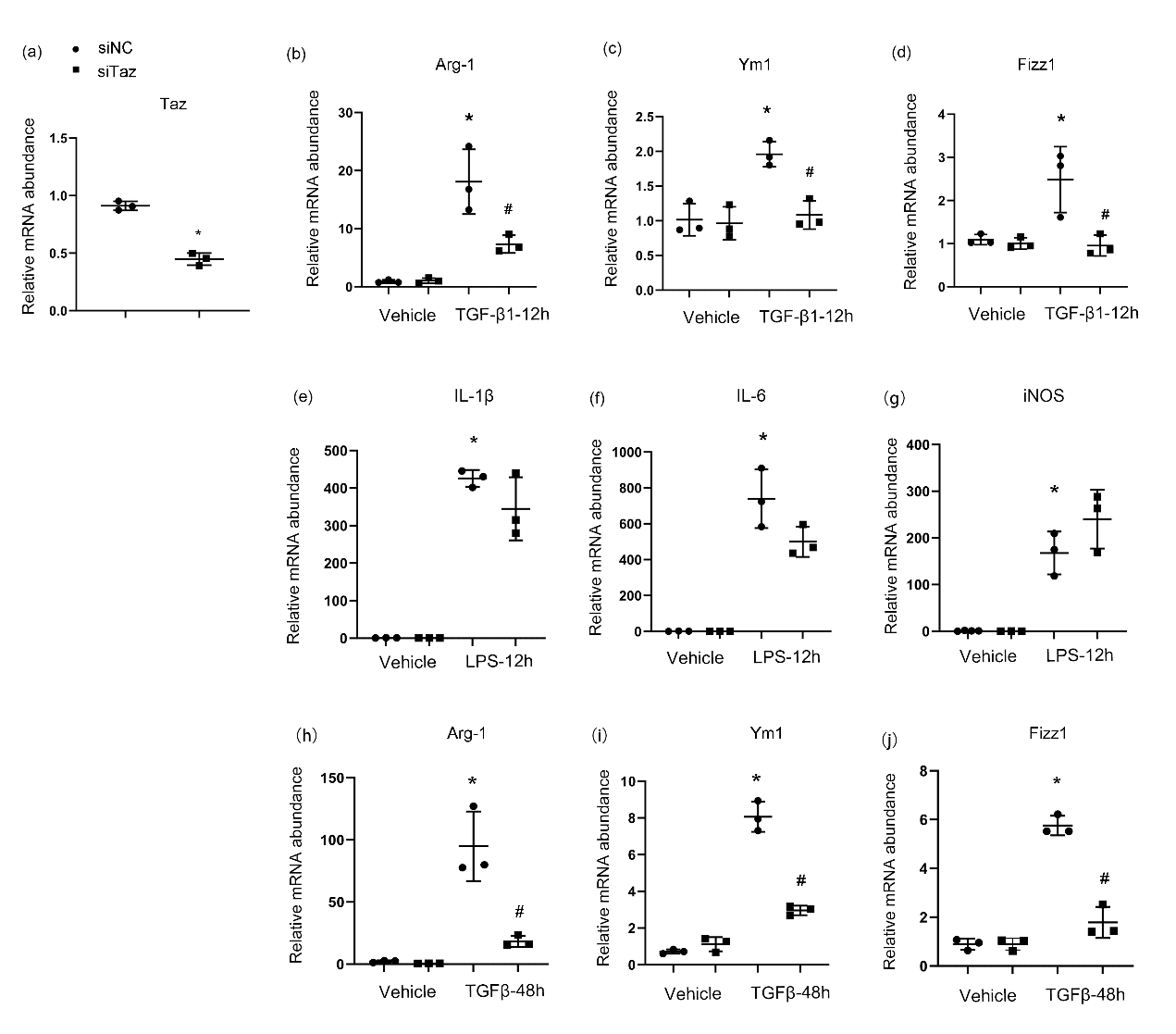

Supplement: Supplementary Materials — The supplementary materials include supplementary Figures 1 and 2 and recording figure legends. [file 9512251.f1.docx]
